# Comparative Analysis of Volatile Compounds in Flowers of Different *Actinidia* Species

**DOI:** 10.3390/plants9121675

**Published:** 2020-11-30

**Authors:** Agnieszka Stasiak, Piotr Latocha

**Affiliations:** Department of Environmental Protection and Dendrology, Institute of Horticulture Sciences, Warsaw University of Life Sciences—SGGW, ul. Nowoursynowska 159, 02-787 Warsaw, Poland; agstasiak@gmail.com

**Keywords:** kiwifruit, kiwiberry, silver vine, lilac compounds, headspace samples, attractants for pollinators, pollination, *Actinidia arguta*, *Actinidia chinensis*

## Abstract

Among the actinidia genus (*Actinidia* spp.) native to China, few species are grown commercially for their edible and healthy fruits. As they are dioecious plants, a lot of interest is paid to effective pollination and to insects as the most efficient pollinators. In this study we have concentrated on the composition of volatile compounds in male flowers of four different actinidia species (*A. chinensis* var. *deliciosa*, *A. arguta*, *A. kolomikta* and *A. polygama*) and on the diversity between male and female flower volatiles for the two most winter-hardy species (*A. arguta* and *A. kolomikta*) with growing commercial value. Analyses were provided using gas chromatography with mass spectrometry (GC-MS). In total, 120 compounds were found in 15 actinidia genotypes. However, the number of identified compounds varied between species. Different main compounds or groups of volatile compounds characterised flowers of every species. Smaller differences were observed between male and female flowers of the same species. Our results suggest that actinidia flowers could be attractive to pollinating insects.

## 1. Introduction

Actinidia genus (*Actinidia* spp.) includes 54 species [1]. However, only few species and varieties are commercially cultivated, including *Actinidia chinensis*, *Actinidia arguta* and *Actinidia kolomikta*. All actinidia species are dioecious woody vines [2] and come mostly from Asia. The most popular species, cultivated worldwide, are *A. chinensis* var. *chinensis* and *A. chinensis* var. *deliciosa* (amounting to 31% and 37%, respectively, of the total actinidia production), but other species are gaining in importance. In particular, *A. arguta*, also known as hardy kiwifruit, kiwiberry or minikiwi, and *A. kolomikta*, known as super-hardy kiwi, amounting already to 17% and 8% of the total output, respectively [3]. *Actinidia arguta*, a smooth-skinned, grape-sized fruit, contains a high amount of vitamin C, phenolic compounds and minerals, representing a high antioxidant activity, and hence a wide range of health benefits [4,5,6,7,8]. *Actinidia kolomikta* is the most tolerant to low temperatures (up to −40 °C). The fruit is smaller than kiwiberry and matures early in the season [2,4]. Generally, *A. arguta* and *A. kolomikta* fruits both present higher levels of antioxidants, vitamin C and concentration of minerals than *A. chinensis* [3]. The fruit of *Actinidia polygama*, which has a nice orange colour and a bitter taste, was used mainly in ancient Chinese and Korean folk medicine [9].

As actinidia are dioecious plants, a lot of attention is given to effective pollination. At first, *A. arguta* was thought to be a wind-pollinated plant. However, previous studies showed that insects like to visit these flowers, and this has a high impact on the fruit set [10,11,12,13]. Moreover, as was found in *A. polygama*, the seemingly perfect (meaning bisexual) flowers (with stamens producing sterile pollen) are more attractive to insects than those with artificially removed stamens [14]. There are also reports that insects are sensitive to specific volatiles and that they visit certain species more on purpose rather than accidentally. Furthermore, many insect species are able to distinguish between enantiomers of different substances, but react only to one. Such behaviour was also found in the case of pollinating insects [15]. Among flower volatile compounds, lilac aldehyde is highly attractive to pollinators (e.g., Lepidoptera) and is emitted in high amounts in nocturnal plant species [16]. Twidle et al. [17,18] found that certain volatiles of both *A. chinensis* varieties are recognized by honey bees and bumble bees. Then, apart from the flowers’ appearance and the number of stamens, the composition of volatile compounds in flowers is also an interesting subject.

To date, more attention is paid to female rather male cultivars, and hence the offer of male selections in nurseries is very low. For various reasons, artificial pollination (often applied in the cultivation of *A. chinensis*) is not practised in kiwiberry production (due to the difficult access to *A. chinensis* and *A. arguta* pollen in most kiwiberry-growing countries and the potential risk of Psa transmission by imported *A. chinensis* pollen). Therefore, the selection of effective pollinizers, both in terms of the pollen quantity and quality as well as the attractiveness of flowers to pollinators, is needed to produce good-quality fruit. Recent research also shows that the source of pollen may have an impact on some biochemical features of kiwiberries and that a proper selection of pollinizers appears to be important in commercial fruit production [19]. Therefore, it would be worthwhile to compare the composition of volatile compounds in flowers of different species of actinidia and to ascertain the diversity within one species.

Volatile compounds in flowers of *A. chinensis* var. *chinensis* and *A. chinensis* var. *deliciosa* have been well analysed, as this species is of great economic importance. An analysis carried out on solvent and headspace samples of cut flowers showed a presence of 107 and 59 compounds, respectively. Most of them were carbonyls, alcohols, hydrocarbons and esters [20]. Another headspace analysis of cut flowers identified 27 mostly terpenoid compounds [21]. Twidle et al. [22] analysed the headspace of unpollinated flowers and identified 45 volatile compounds, mostly straight chain hydrocarbons and terpens.

To date, little attention has been paid to volatile compounds of flowers of other actinidia species. The chemical analysis (headspace and solvent extracts GC-MS (gas chromatography—mass spectrometry)) of seven female and two male genotypes of *A. arguta* helped to identify some 60–70 compounds in flowers and 40–80 in fruits. The most numerous chemical groups in flowers were represented by terpenes (including lilac derivatives coming from linalool) and benzenoid compounds. A GC analysis showed differentiation in the composition of flower volatile compounds between the tested genotypes [23]. A study on a different species has shown that the flower aroma of even closely related species, such as *Brunfelsia australis* and *B. pauciflora*, may differ significantly [24]. Considering the above, understanding the composition of volatile compounds in flowers of less known species of actinidia, may help to optimize the commercial production of their fruit.

The aim of the research was to compare the composition of volatile compounds of flowers from male genotypes of *A. chinensis* var. *deliciosa*, *A. arguta*, *A. kolomikta* and *A. polygama*, as well as male and female genotypes of *A. arguta* and *A. kolomikta*, the two most winter-hardy actinidia species commercially cultivated in colder regions.

## 2. Results and Discussion

### 2.1. Composition of Volatile Compounds in Actinidia Flowers

Flowers of tested species differ in size. *Actinidia chinensis* var. *deliciosa* produce the biggest flowers (up to 4 cm in diameter), while *A. kolomikta* produce the smallest (usually ~1.5 cm in diameter). The structure of flowers appears to be less diverse. However, male and female flowers differ significantly. Male flowers contain only numerous stamens, while the female ones, seemingly perfect, have—in addition to residual stamens (producing empty, non-viable pollen)—a pistil with radially arranged styles.

**Figure 1:**
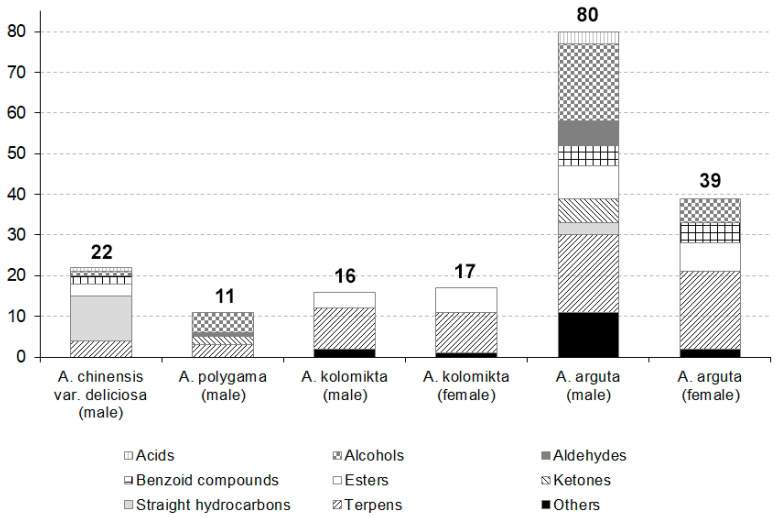
Differences in number and composition of volatile compounds in particular actinidia species.

**Figure 2:**
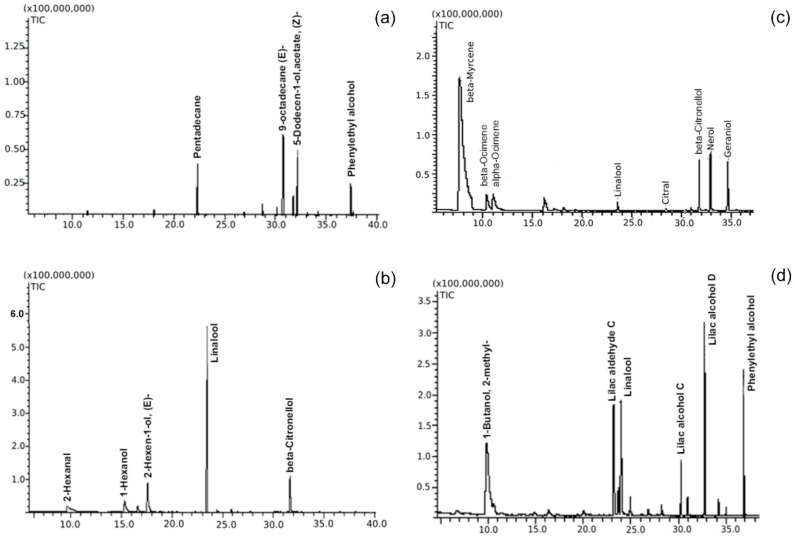
Sample chromatograms with main volatile compounds in male flowers of four actinidia species: (**a**) *Actinidia chinensis* var. *deliciosa*; (**b**) *Actinidia polygama*; (**c**) *Actinidia kolomikta*; (**d**) *Actinidia arguta*.

In total, 120 different volatiles, which can be divided into nine groups, were found. About 22 compounds were found in flowers of *A. chinensis* var. *deliciosa*, 16–17 in *A. kolomikta* (male and female, respectively), about 80 and 39 in *A. arguta* (male and female, respectively) and merely 11 compounds in *A. polygama* flowers (Figure 1). Significant differences were observed between species in the composition of volatile compounds in flowers. Hydrocarbon 9-octadecene E-dominated in *A. chinensis* var. *deliciosa*. *A. arguta* was characterised by the presence of linalool and its derivatives. Beta-myrcene was the main compound (>65%) in *A. kolomikta*, while linalool constituted almost half of the emitted volatiles in *A. polygama* (Table 1, Figure 2a–d). All identified compounds can be divided into chemical groups dominating in the particular species (Figure 1). *A. kolomikta* and *A. arguta* were characterised by a high diversity of terpens, while only few terpenoid compounds were found in *A. chinensis* var. *deliciosa* and *A. polygama*. However, the *A. chinensis* var. *deliciosa*’s headspace sample was characterised by a significant number of straight-chain hydrocarbons. In *A. polygama* and *A. arguta* plenty of alcohols were identified, but with a difference between these two species (Table 1, Figure 2b,c). Considering all volatiles in these species, the most numerous groups were alcohols, terpens, esters and straight hydrocarbons, amounting to about 26, 21, 13 and 12% of all the identified volatiles, respectively. Among terpens, the largest group consisted of linalool derivatives identified in all tested *A. arguta* genotypes. On the other hand, ketones, aldehydes and acids were not typical for most of the tested species, and certain groups were observed in just one or two species.

### 2.2. Differences in the Composition of Volatile Compounds in Flowers of Different Actinidia Species

In our research, much of the *A. chinensis* var. *deliciosa* headspace samples were straight-chain hydrocarbons. None of these were found in other tested species (Table 1). Two of these, 9-octadecene (E) and pentadecane, were identified as the main compounds and accounted for 31.8 and 19.6%, respectively (Figure 2a), of the total. These two compounds were previously identified in *A. chinensis*. 9-octadecene (E) was found in *A. chinensis* var. *deliciosa* cultivars, yet in a much smaller percentage (0.03–0.13%) [22]. As in previous research, a similar level of pentadecane was found in *A. chinensis* var. *chinensis* and in *A. chinensis* var. *deliciosa* (9–20%, depending on the cultivar) [20,22]. 5-dodecen-1-ol, acetate, (Z)- (19.9%) and phenylethyl alcohol (9.2%) represented other important compounds. The latter (phenylethyl alcohol) was also found in *A. arguta* male (0.2–12.2%) and female (5.3–25.0%) flowers. A few more aromatic straight-chain hydrocarbons—heptadecane (3.0%), hexadecane (1.8%), and nonadecane (0.7%)—were also found in the *A. chinensis* headspace. Finally, 2-phenethyl acetate (0.4%), and benzoic compound—1,3-dimethylbenzene (0.4%) were identified in the *A. chinensis* var. *deliciosa* sample. *A. chinensis* var. *deliciosa* headspace was characterised by the presence of a few terpens. These were: alpha-Farnesene, ocimene, cis-Geranylacetone and D-limonene (the one with a well-known lemon-like odour). Their content in headspace amounted to 5.0, 1.2, 0.4 and 0.4%, respectively. D-limonene was also found previously in *A. chinensis* flowers [20,21].

Comparison to previous research on both *A. chinensis* varieties indicated that the particular genotypes differed in the number of identified compounds, composition and amount [17,18,20,21,22]. Five of the compounds identified by Twidle et al. [18] in *A. chinensis* var. *deliciosa,* namely alpha-Farnesene, hexadecane, cis-geranylacetone, nonadecane and phenylethyl alcohol, were also found in our research. Similar compounds such as those in our research, namely terpens (D-limonene, ocimene, alpha-Farnesene), straight-chain hydrocarbons (pentadecane) and alcohols, like phenylethyl alcohol, were found by Tatsuka et al. [20]. Among the 27 compounds identified by Samadi-Maybodi, Shariat and Zarei [21] in *A. chinensis*, only three (limonene, ocimene and alpha-Farnesene) were identical to the ones in our research. The following compounds were found in our and previous research in *A. chinensis* var. *deliciosa* flowers: tridcane, nonadecane, pentadecane, hexadecane, 7-hexadecene, heptadecane, alpha-Farnesene, 2-phenylethyl acetate, phenylethyl alcohol and cis-geranylacetone. However, the latter compound was found in one of the six genotypes of *A. chinensis* var. *deliciosa* [22]. Alpha-Farnesene was the one compound found in all available results; however, its percentage of all the identified compounds stood between 0.6 and 38.0%, depending on the information source [17,18,20,21,22]. No linalool and lilac compounds characteristic of *A. arguta* were found in *A. chinensis* [23,25].

When analysing the *A. polygama* flower aroma in detail, it was found that some compounds were characteristic of that species only. These included, e.g., 2-hexenal (6.6%) and 2-hexen-1-ol, (E)- (17.5%) (a primary allylic alcohol, used for food flavouring, PubChem). Form (Z)- was also found, but in a much smaller amount (0.2%). However, linalool (47.8%) (Figure 2b), the major compound of *A. polygama* volatiles, was also discovered in eight out of eleven *A. arguta* samples. Beta-citronellol (13.2%) was another important compound of *A. polygama* also found in *A. kolomikta*, yet in a minor amount (3.5%) (Figure 2b,c). Generally, only few volatiles of *A. polygama* were found in other species. Along with the already mentioned linalool and beta-citronellol, 3-hexen-1-ol, (Z) (4.5%) and 1- hexanol (8.6%) were identified. Both were also found in two male *A. arguta* genotypes (‘Rot’, ‘Rubi’). Additionally, 1-hexanol was present in ‘Nostino’ and ‘Joker’. It is worth noting that no common volatile compounds for *A. chinensis* and *A. polygama* flowers were identified.

In *A. kolomikta*, monoterpene β-myrcene was the compound representing the highest relative content (~75% in male and ~67% in female) (Table 1, Figure 2c). Myrcen, an isomer of ocimene, is said to be unstable in the air and tends to polimerate easily [26] (p. 280). Nerol and geraniol (4.0% and 3.4%, respectively), identified in male *A. kolomikta*, turned out to be characteristic of this species alone and were not traced in other actinidia species. Another characteristic feature of *A. kolomikta* compounds were citral isomers (geranial and neral), responsible for the lemon note in the aroma. Neral accounted for less than 1%, yet its aroma is very strong. Certain volatiles were found not only in *A. kolomikta*, but also in other actinidia species. Citronellol was one of them, mentioned above, while the others were terpens ocimene α and β, found in *A. kolomikta* and *A. arguta*. Beta-ocimene was also identified in *A. chinensis* var. *deliciosa*’s flowers. Other substances found in *A. kolomikta* and in a few *A. arguta* genotypes were alpha-Farnesene, 1,4-Hexadiene, 5-methyl-3-(1-methylethylidene) and a group of esters, with each of these accounting for less than 1%. Alpha-Farnesene was also found in *A. chinensis* var. *deliciosa*.

Summing up the above, it appears that the number of volatile compounds and their composition differ in the particular actinidia species. Moreover, every species seems to have one or few dominating compounds affecting their flower aroma.

### 2.3. Diversity of Volatile Compounds in Flowers of Different A. arguta Genoptypes

**Figure 3:**
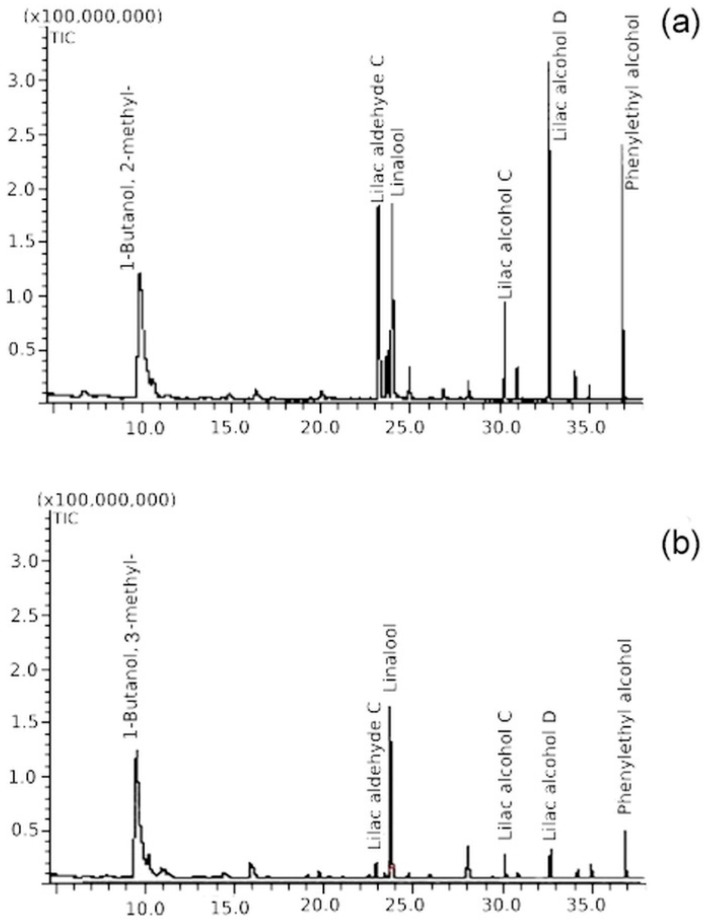
Sample chromatograms with main volatile compounds in flowers of two different male *Actinidia arguta* genotypes (**a**) “Nostino” (**b**) “Haya Kume”.

*Actinidia arguta* is the species with the largest area of natural occurrence, both in terms of latitude and altitude [27]. This affects the large morphological diversity within the species (e.g., in terms of leaf shape, size, shape and colour of the fruit), which may account for the richest composition of flower volatile compounds, belonging to at least eight different chemical groups. Among the 120 detected compounds, about 80 were found in seven male clones of *A. arguta* (Table 1, Figure 3). Of these, ten (lilac aldehydes, lilac alcohols, 5-Hepten-2-one, 6-methyl-, geranyl acetone, phenylethyl alcohol, 1,7-Octadien-3-ol, 2,6-dimethyl-, 1,4-Hexadiene and 5-methyl-3-(1-methylethylidene)) were detected in all seven male clones. Their relative content varied depending on the genotype, even in the same sex (Figure 3). However, about 44 compounds (55%) were detected only in one genotype, and nine in two. These were mainly esters, ketones, alcohols and straight-chain hydrocarbons. Few terpenoid volatiles, excluding linalool and ocimene derivatives, were found only in one or two genotypes. The relative content of these compounds was usually small and accounted for less than 1%.

In this research, linalool derivatives represented the most important group found in *A. arguta* flowers. Linalool, lilac aldehydes and alcohols were present in almost all samples (Figure 2d). Differences in their composition in headspace samples were high, and their summed content varied between 24.3% and 92.7%. A detailed analysis showed that in few instances more than one peak was identified as representing the same compound (among lilac aldehydes and alcohols). That was probably due to not distinguishing isomers by mass spectrometer. The analysis of retention indices was not conclusive either, as only partial information on the compounds analysed is available in the literature and online data. An additional detailed analysis is needed to clarify whether these peaks are indeed two isomers of the same compounds. When summing up all lilac aldehydes for each genotype, it follows that differences were high. The lowest percentage of the compound was found in ‘Haya Kume’ (2.7%), while the headspace of the other clones contained at least 17.0% (‘Weiki’). In the *A. arguta* ‘Rot’, which was previously classified as *A. arguta* var. *purpurea* (form with red fruit), the content of lilac aldehyde amounted to 25.0%. According to the latest genetic revision [1], *A. arguta* var. *purpurea* has been included in the *A. arguta* variability. The highest content was observed in flowers of clone F7 (53.0%), which is a hybrid between *A. arguta* and *A. arguta* var. *purpurea*. This may suggest, however, that the offspring of this cross might produce a higher amount of lilac aldehyde in flowers. The analysis of lilac alcohol led to a similar observation. The highest percentage of the compound was observed for F7 (39.7%) and the lowest for ‘Haya Kume’ (5.4%). *Actinidia arguta* ‘Rot’ represented a rather low content of lilac alcohol in flowers (12.9%). The sum of all isomers of lilac aldehyde and alcohol ranged between 38 and 53% for most of the tested genotypes. The lowest content of these compounds in the headspace has been ascertained in the ‘Haya Kume’ sample (8.1%), which also had the least intense aroma, organoleptically (own observation, unpublished). The content of lilac alcohol and aldehyde isomers exceeded 92% in the F7 genotype. It is noteworthy that in two male samples, ‘Weiki’ and F7, no linalool was detected. Isomers of linalool derivatives in *A. arguta* were identified by Dötterl et al. [16]. The authors found two stereoisomers of linalool (S, R), four of lilac aldehydes and four of lilac alcohol. The same combination of stereoisomers was found in two out of 15 species investigated (*Cynanchum auriculatum* and *Viburnum opulus*). Some species contained one stereoisomer of linalool and the same (*Syringa vulgaris*) or similar (e.g., *Silene otites*, *Prunus padus* and *Daphne cneorum*) combination of lilac alcohol and aldehyde stereoisomers. Further analysis revealed that derivatives of linalool were more complex than expected. In earlier research of *A. arguta*, these derivatives were present only in a few examined genotypes, and some of these were found in solvent extracts [23,25]. When investigating the stereoisomers of lilac aldehyde, it appears that our results differ from those by Matich et al. [23], where only one headspace male flower sample missed one lilac aldehyde stereoisomer, whereas in this particular study only lilac aldehydes C and D were found. However, as observed earlier, the problem could be due to an inappropriate distinction of lilac stereoisomers. In a previous study, four stereoisomers of lilac alcohol were also found in headspace samples [23], while only two forms were found in our analysis. A comparison to the insightful research undertaken by Matich et al. [23] shows that lilac compounds, which were first isolated from *Syringa vulgaris* flower oil and detected in at least nine plant families [16], are important components of *A. arguta* headspace samples. In our analysis, however, the relative content of both lilac alcohol and aldehyde was higher than in Matich et al.’s [23] analysis. The reason may be methodological (that is, the use of a different method of collecting samples) as well as genetical. 5-Hepten-2-one and 6-methyl were also detected by Matich et al. [23] in all headspace and solvent samples, but the content amounted to less than 1% in all genotypes.

Ocimene derivatives constitute another important group among terpens. In this work, E-beta ocimene was identified in most samples, and its relatively highest content (7.0%) was found in ‘Haya Kume’. Geranylacetone (found, e.g., in *Nelumbo nucifera* oil) was present in all clones and 5-Hepten-2-one, 6-methyl- (a component of volatile oils of citronella, lemon-grass and palmarosa oil) in most (e.g., 4.9% for ‘Rubi’). 5-Hepten-2-one and 6-methyl were also detected by Matich et al. [23] in all headspace and solvent samples, but the content amounted to less than 1% in all genotypes. Other terpens identified in this analysis were previously found only in single samples. However, alpha-Farnesene, detected here in the headspace, was earlier found only in the solvent extract of one female genotype.

Only few benzoid compounds were identified in the headspace samples. One was the already mentioned phenylethyl alcohol. The highest content was noticed in ‘Joker’ (12.2%), but it stood below 5% in most of the clones. Two benzoid compounds (e.g., 2-(4-Methoxyphenyl) ethanol) were found in six out of seven male clones, but their content was low (< 0.5%). Benzeneacetaldehyde, which is an aromative compound, was identified in four clones, but its content was low as well (< 1%). The results of Matich et al. [23] suggest that benzoid compounds are not common in the headspace, but high amounts are present in solvent samples (up to 30.1%). The only compound, identified by them in larger amounts in volatile samples, was phenylethyl alcohol (6.25 and 17.2%). That was confirmed in our research. 2-(4-Methoxyphenyl) ethanol was also identified by Matich et al. [23], yet only in solvent extracts. It was found there in small amounts but in almost all samples. Nor was benzeneacetaldehyde found in previous studies, but benzaldehyde and benzene (the compounds that were not found in our genotypes) were detected in several samples. This suggests that among benzoid compounds only phenylethyl alcohol is specific to *A. arguta*, in male and female genotypes alike. The identification of other compounds may depend on their concentration in the headspace, detection possibilities, genotype or environmental impact.

More than ten ester compounds were identified in all samples of *A. arguta*. Of these, butanoic acid, 3-methyl- and 3-methylbutyl ester, a constituent of banana fruit, tomatoes and alcohol beverages (e.g., cognac or cider), was found to be the compound that may have an impact on the aroma. The ester was found in four male samples. The content in two (‘Nostino’ and ‘Haya Kume’) amounted to less than 0.5%, and in other two (‘Rot’ and ‘Weiki’) to 2.1 and 3.3%, respectively. More esters were found in this research than in previous ones in general. Reports of using SMPE sampling for the analysis of fatty acid esters, whose particles are heavier than those identified in our research [28], suggest that the sampling methods used contributed to a better ester detection in this research.

Only three aldehydes (furfural, 5-methylfurfural and nonanal) were detected in this research. Furfural and 5-methylfurfural were present in small amounts in the ‘Nostino’ sample (1.3% and 0.3%, respectively) and nonanal in ’Joker’ (0.1%), whereas Matich et al. [23] identified 11 different aldehydes. Four, including nonanal, were present in all samples. Ketones were detected in two male genotypes: ‘Nostino’ and ‘Haya Kume’, but the amounts of particular compounds were different. Most of them also amounted to less than 1% of the total headspace, but the contents of one ketone—3-Octanone, detected in ‘Haya Kume’—were higher and reached 4.2%. The analysis of ketones in both studies shows that genotypes tend to have individual combinations of these compounds or almost no ketones.

High differences in relative content were also noticed for two alcohols: 1-Butanol, 2-methyl- and 1-Butanol, 3-methyl. Genotypes with a high content of lilac compounds (F7) presented a low content of these alcohols (1.5% in F7 headspace); and, conversely, where a low content of lilac compounds was found, a high percentage of these alcohols was observed (up to 49.4% in ‘Haya Kume’). These two alcohols were also found by Matich et al. [23] in the headspace of *A. arguta* samples. In our and Matich et al.’s [23] research, one or the other compound was present in the sample, but never both of them simultaneously. 1-Octen-3-ol was also found in previous research, but only in two genotypes. Generally, few alcohols detected in our research were also found by other researchers, but a few different compounds were also identified. Some were chemically similar, e.g., in terms of the same base, but different in the location of a double chemical bond or substituent. This usually has a significant impact on a compound’s characteristics. Straight-chain hydrocarbons, like acids, were rather uncharacteristic for *A. arguta* flower volatiles. As in our analysis, Matich et al. [23] detected only few hydrocarbons, and most were found in just one genotype. In this work, only a few acid compounds were found, and they do not seem to have an impact on the aroma of *A. arguta* flowers.

Summing up, lilac compounds are the most important compounds in most *A. arguta* male genotypes. Alcohols also play an important part in the aroma, but a number of these substances were found only in a few or just one genotype. Some alcohols represent regular components of the aroma but differ in their relative content. Moreover, every genotype/variety has its own unique volatiles composition. This offers the possibility of selecting genotypes with a higher content of volatile compounds, and enhances their attractiveness for pollinating insects for actinidia commercial orchards.

### 2.4. Male Versus Female A. arguta and A. kolomikta Genotypes Comparison

**Figure 4:**
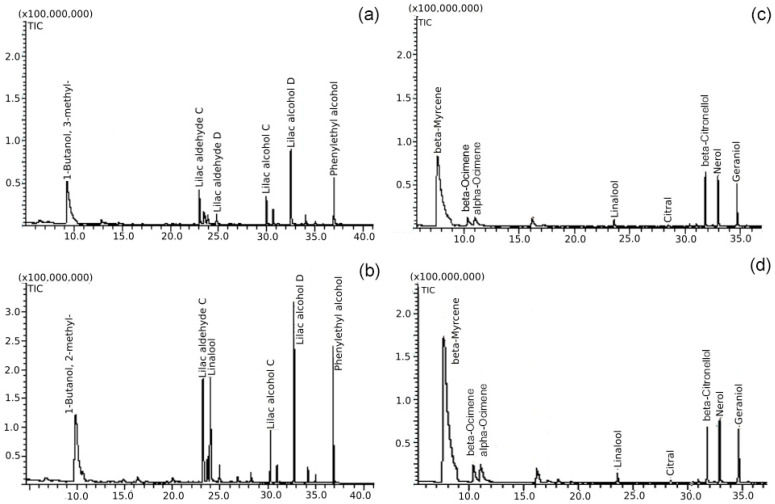
Sample chromatograms with main volatile compounds in flowers of female and male *Actinidia arguta* (**a**,**b**) and *Actinidia kolomikta* (**c**,**d**) genotypes: (**a**) “Ananasnaya”, (**b**) “Nostino”, (**c**) female, (**d**) male.

*Actinidia arguta*’s female genotypes were characterised by a composition of volatiles similar to that of male ones (Figure 4a,b). However, less compounds (39) were detected in female ones. The reason could be due to the smaller number of genotypes, considering that most compounds in male samples were detected in only one genotype. Among female genotypes, important compounds were also represented by lilac compounds (Table 1). Their overall content varied between 29.5% for ‘Geneva’ and 85.4% for the ‘Bingo’ cultivar. The latter figure was much higher than the one observed in other varieties. As ‘Bingo’ is a crossing of *A. arguta* male and *A. arguta* with red fruit (previously classified as *A. arguta* var. *purpurea*), our previous observation of male genotypes was confirmed to the effect that such combination represents a higher content of linalool derivatives. In our analysis, as in that of Matich et al. [23], a higher percentage of linalool was observed in male genotypes. A relatively higher content of phenylethyl alcohol was noted in female than in male genotypes.

Interestingly, different esters were identified in flowers of male and female genotypes (Figure 4c,d). Only one ester (butanoic acid, 3-methyl-, 3-methylbutyl ester) was detected in both sexes, but still not in every genotype. No aldehydes, ketones or acids were found in female *A. arguta* genotypes. The composition of alcohols was smaller in female flowers, but high amounts of 1-Butanol and 3-methyl (main alcohol of male flowers) were also found in female genotypes.

A comparison of male and female clones of *A. kolomikta* showed almost no differences. Two compounds found in a female clone—that is, 9-Octadecenoic acid (Z), methyl ester, and 12,15-Octadecadienoic acid, methyl ester—were not detected in the male one. On the other hand, one compound, namely furan 3-(4-methyl-3-pentenyl), was found only in male flowers. The differences between sexes in *A. kolomikta* were observed in the relative content of the main compound, beta-myrcene (e.g., 74.7% in the male and 67.2% in the female clone), and in a few other aromatic terpens, like beta-citronellol, trans-geraniol and nerol (Table 1).

Summing up, the female flowers of *A. arguta* may have a simpler composition of volatiles than the male ones, but the observation of *A. kolomikta* suggests that two randomly chosen genotypes of this species may be very similar to each other.

### 2.5. Pollinators’ Interest

Since actinidia is dioecious, effective pollination is essential to obtain well-formed fruit. Honey bees seem to be the most important pollinators for kiwifruit [29]. Bumble bees are also used for pollination, especially since they are commercially available [22]. Our unpublished observation of kiwiberry orchards in Polish conditions suggests that *A. arguta* flowers are visited mainly by *Bombus terrestris*, *B. lapidarius* and *Apis mellifera*. According to Twidle et al. [18], honey bees and bumble bees are interested in certain *A. chinensis* volatile compounds from male and female flowers. Some of these compounds also found in our research, e.g., phenylethyl alcohol, alpha-Farnesene, linalool, 6-methyl-5-hepten-2-one, geranyl acetone, 2-phenylethyl acetate and 8-heptadecene, were the same or similar isomers found in at least one sample of *A. arguta* flowers in our research. Phenylethyl alcohol and 6-methyl-5-hepten-2-one were present in most samples. The first compound, as Twidle et al. [17,18] found out, provided the largest response from antennae in both honey and bumble bees. Furthermore, seven out of 19 compounds attractive to the investigated pollinators were found in our *A. chinensis* var. *deliciosa* sample. Honey bees were observed to respond to phenylethyl alcohol, 1-hexanol and (Z)-3-hexen-1-ol. These compounds were also attractive to nocturnal bees—major pollinators for cambuci plants (*Campomanesia phaea*) [30]. In other reports, linalool was observed to attract nocturnal bees *Megalopta* on guarana (*Paullinia cupana*) [31], and honey bees on *Medicago sativa* [32] and pears (*Pyrus communis*), where (E)-β-ocimene and lilac alcohols were also found to be attractive to bees [33]. Compounds like α-pinene and D-limonene were also found to be attractive to *A. mellifera* [34], with the second one identified in the *A. chinensis*’ headspace sample.

A comparison of our data on volatile compounds in actinidia flowers and the literature about insect interest allows us to conclude that actinidia flowers may be attractive to pollinating insects, which makes it possible to select the best genotypes, especially male ones.

## 3. Materials and Methods

### 3.1. Plant Material

The experiment was carried out in 2019. Mature plants of each genotype were grown in the Experimental Garden of the Environmental Protection and Dendrology Department at the Warsaw University of Life Sciences, in central Poland. Flowers of the following genotypes were analysed: male—*A. chinensis* var. *deliciosa*, *A. polygama*, *A. kolomikta* and *A. arguta*; female—*A. kolomikta* and *A. arguta.* The last species was represented by four female and seven male genotypes (Table 2).

### 3.2. Identification and Determination of Volatile Compounds

#### 3.2.1. Collecting Flowers and Volatile Samples

Twenty-five flowers of each genotype were collected from just opened flowers in the middle of the full flowering period. They were then put in 250 mL glass bottles, transported immediately to the lab and kept in ambient temperature for an hour before analysis. An exception was *A. chinensis* var. *deliciosa*, where only five flowers were collected due to the flower size.

#### 3.2.2. Determination of Volatile Compounds

GC-MS was used for the identification and determination of volatile compounds. The GC-MS analyses were carried out using a mass spectrometer coupled with a gas chromatograph (GCMS-QP2010, Shimadzu, Japan) fitted with a Stabilwax column (crossbond carbowax polyethylene glycol phase) (30 m; 0.25 mm i.d.; 0.25 mm film thickness). The procedure was based on Bertrand, Comte and Piola [24], developed for *Brunfelsia* sp. flowers, with some modifications. The oven temperature was programmed from 50 °C to 250 °C at 3 °C/min; the injector temperature was 250 °C; the carrier gas, helium, was adjusted to a constant flow of 1 mL/min; injection type: splitless. EI-MS: electron energy, 70 eV; ion source temperature and connection parts: 250 °C. Headspace volatiles were collected using the SPME (DVB/CAR/PDMS) fibre (Sigma-Aldrich, Darmstadt, Germany). The fibre was put into the bottle for 20 min at room temperature and for the following 2 min in 250 °C in a GC injector. The identification of volatile compounds was made on the basis of mass spectral libraries of the National Institute of Standards and Technology (NIST 47, NIST 147) and Wiley 175, as well as data from the literature [23]. Retention indices were calculated according to van Den Dool and Kratz [35], dedicated to a linear temperature programmed GC. The calculations were based on the retention times of saturated alkanes C7–C30. Retention indices were calculated for the compounds with retention time ranging between 20.965 and 56.909 min, and compared to data available online at https://webbook.nist.gov/. The peak shapes of saturated alkanes below 20.965 and after 56.909 min were not satisfactory as they lacked a clear central value, and prevented the accurate determination of their retention times. In consequence, the calculation of the retention indices of volatile compounds was not possible, and additionally, retention times for all compounds were added to the Table 1. If the similarity to mass spectra libraries was below 90% and the comparison of retention indices was not possible, the most abundant six mass spectral ion fragments were also listed in the table in descending order. For important compounds, ten fragments were reported. The results were rounded to one decimal place and presented as relative percentages of peak areas of the total ion count in the samples, separately for each genotype.

## 4. Conclusions

Summing up the above, 120 volatile compounds were found in total. The biggest group were terpens and alcohols—31 and 25 compounds, respectively. The highest number of volatiles were found in *A. arguta* genotypes, about 80 compounds were found in seven male genotypes (but 44 compounds were found only in a single genotype, and 10 in all), and about 39 in four female genotypes. In other species, less volatiles were found: male *A. chinensis* var. *deliciosa*—22, *A. kolomikta*—17 in the female and 16 in the male genotype, and *A. polygama* male—11 compounds. A high diversity of volatile compounds was observed between species, e.g., *A. polygama* and *A. chinensis* var. *deliciosa* had no common substances. In all species, a characteristic compound or group of compounds could be found. However, differences within species were not so clear, e.g., the composition of volatiles in *A. kolomikta* was very similar in both sexes, but volatiles of *A. arguta* genotypes were more diverse, and this was more related to the plants’ origin than their sex.

Considerable differences in the composition of volatile compounds in male A. arguta genotypes indicate their possible selection in terms of attractiveness to pollinating insects, regardless of other values. The selection of effective pollinators in commercial kiwiberry cultivation can increase yields and produce better-quality fruit. Therefore, further and more detailed studies on the content of volatile compounds in flowers of various species of actinidia and their influence on the behaviour of pollinating insects are needed.

## Figures and Tables

**Table 1:** Volatile compounds identified in flowers of four actinidia species (all genotypes tested). Data represent the approximate relative percentage of peak areas for each given species. **Bold name** = mass spectra library match strength 90% or greater, **Bold Retention Indices (RI)** = positively compared to Van Den Dool and Kratz RI on https://webbook.nist.gov/.

Compound	Retention Time	Retention Index *	Retention Index, Literature Data **	*A. chinensis* var. *deliciosa*	*A. polygama*	*A. kolomikta*		*A. arguta* Male Genotypes							*A. arguta* Female Genotypes			
				Male	Male	Male	Female	‘Nostino’	‘Joker’	‘Rot’	‘Weiki’	‘Rubi’	‘Haya Kume’	F7	‘Geneva’	‘Bingo’	‘Anna’	‘Weiki’
Terpens																		
**Geranial**	28.451	1637	-	-	-	0.2	0.3	-	-	-	-	-	-	-	-	-	-	-
**Neral**	29.451	**1660**	1660	-	-	0.2	0.4	-	-	-	-	-	-	-	-	-	-	-
**beta-Citronellol**	31.677	1737	1755	-	13.2	3.5	6.3	-	-	-	-	-	-	-	-	-	-	-
**D-Limonene**	9.114	-	1190	0.4	-	-	-	-	-	-	-	-	-	-	-	-	-	-
Eucalyptol (43, 32, 81, 71, 84, 69 ***)	8.812	-	1220	-	-	-	-	-	-	-	-	-	-	-	-	0.3	-	-
**Alpha-Farnesene**	31.732	**1739**	1740	5.0	-	0.2	0.4	-	-	0.5	-	-	-	-	-	-	0.3	-
**trans-Geraniol**	34.680	**1833**	1833	-	-	3.4	5.1	-	-	-	-	-	-	-	-	-	-	-
**5-Hepten-2-one, 6-methyl-**	14.895	-	1323	-	-	-	-	0.9	2.9	2.6	1.3	4.9	1.2	0.5	-	0.2	0.4	0.4
Lilac aldehyde B (55, 43, 41, 67, 93, 29, 71, 27, 69, 111)	22.855	1462	-	-	-	-	-	-	-	-	-	-	-	-	1.0	-	-	14.4
Lilac aldehyde C (55, 43, 41, 67, 93, 71, 27, 29, 111, 39)	23.199	1473	-	-	-	-	-	12.8	12.9	9.8	7.8	19.9	1.6	13.4	-	13.7	8.5	4.3
Lilac aldehyde C (55, 43, 41, 71, 67, 93, 29, 27, 69, 68)	23.671	1489	-	-	-	-	-	3.35	4.6	5.0	3.5	6.4	0.7	7.8	-	4.4	2.8	-
Lilac aldehyde D (55, 43, 41, 71, 67, 93, 29, 27, 69, 68)	24.615	1519	-	-	-	-	-	1.7	3.4	10.3	3.4	3.2	0.4	11.2	-	6.1	2.1	3.7
Lilac aldehyde D (55, 43, 41, 71, 67, 93, 29, 69, 27, 39)	24.947	1529	-	-	-	-	-	-	-	-	2.3	-	-	20.6	-	12.7	2.3	3.4
**Linalool**	23.987	1499	1548	-	47.8	0.7	1.0	11.5	21.8	22.8	-	16.8	16.2	-	-	4.4	1.6	5.9
Lilac alcohol C (55, 43, 111, 93, 67, 41, 71, 69, 29, 81)	29.696	1675	-	-	-	-	-	4.0	4.0	3.8	7.6	2.8	0.5	18.6	2.8	14.0	5.7	2.0
Lilac alcohol C (55, 43, 111, 93, 41, 67, 71, 29, 69, 81)	30.276	1693	-	-	-	-	-	1.4	2.9	5.1	4.5	1.3	0.6	2.0	14.0	17.6	2.9	1.2
Lilac alcohol C (55, 43, 111, 93, 67, 41, 71, 69, 29, 81)	30.967	1714	-	-	-	-	-	1.0	1.4	1.0	1.9	0.9	-	-	2.6	2.9	1.8	-
Lilac alcohol C (55, 43, 93, 111, 41, 67, 71, 69, 29, 81)	32.197	1754	-	-	-	-	-	-	12.0	-	-	-	-	-	-	-	-	-
Lilac alcohol D (55, 43, 93, 111, 67, 41, 71, 69, 29, 81)	32.754	1772	-	-	-	-	-	13.8	-	3.0	12.5	10.3	1.7	6.1	9.1	9.7	14.2	4.0
Lilac alcohol D (55, 43, 93, 111, 67, 41, 71, 69, 29, 81)	33.700	1802	-	-	-	-	-	-	-	-	-	-	2.6	12.9	-	-	-	9.5
Lilac alcohol D (55, 43, 93, 111, 41, 67, 71, 69, 81, 29)	34.228	1820	-	-	-	-	-	-	-	-	-	-	-	-	-	-	-	9.7
**linalyl oxide**	20.602	-	1468	-	0.6	-	-	0.1	-	-	-	0.2	0.6	-	-	0.1	-	-
**beta-Myrcene**	7.649	-	1148	-	-	74.7	67.2	-	-	-	-	-	-	-	-	-	-	-
Cis-myrtanol (41, 121, 67, 55, 29, 79)	17.233	-	-	-	-	-	-	-	0.4	-	-	-	-	-	-	-	-	-
**Nerol**	32.960	**1778**	1777	-	-	4.0	5.8	-	-	-	-	-	-	-	-	-	-	-
**alpha-ocimene**	11.362	-	1232	-	-	3.9	3.6	-	0.4	0.3	-	0.4	-	-	-	-	-	0.1
**E-beta-ocimene**	11.946	-	1230	1.2	-	3.9	3.5	2.8	2.2	2.1	-	1.6	7.0	0.3	-	0.4	-	-
1,6-Octadiene, 3,5-dimethyl-, trans- (69, 41, 39, 79, 53, 82)	13.651	-	-	-	-	-	-	-	-	1.7	-	-	-	-	-	-	0.5	0.3
**5,9-Undecadien-2-one,** **6,10-dimethyl-, (E)-**	34.002	**1842**	1850	-	-	-	-	0.6	1.1	2.2	0.7	1.7	0.9	1.2	0.9	0.5	0.7	0.7
**5,9-Undecadien-2-one,** **6,10-dimethyl-, (Z)-**	35.478	**1857**	1855	0.4	-	-	-	-	-	-	-	-	-	-	-	-	-	-
**Trans-Linaloloxide**	19.432	-	-	-	-	-	-	0.2	-	-	-	-	-	-	-	-	-	-
Benzenoid compounds																		
Benzaldehyde (77, 106, 105, 51, 50, 52)	22.335	1445	1504	-	-	-	-	-	0.1	-	-	-	-	-	-	-	-	0.2
Benzene, 1-(dimethoxymethyl)-4-(1-methoxy-1-methylethyl)-(193, 209, 97, 135, 165, 45)	22.807	1460	-	-	-	-	-	-	0.1	0.4	0.2	0.3	0.3	0.3	-	-	0.2	-
**Benzene, 1,3-dimethyl-**	6.998	-	1140	0.4	-	-	-	-	-	-	-	-	-	-	-	-	-	-
**Benzeneacetaldehyde**	26.797	1586	1617	-	-	-	-	0.6	0.9	0.3	-	0.7	-	-	-	-	-	1.9
**2-(4-Methoxyphenyl) ethanol**	49.788	-	-	-	-	-	-	0.3	0.2	0.4	0.3	0.6	0.4	-	2.1	0.6	0.5	0.5
**Phenylethyl Alcohol**	36.864	**1900**	1902	9.2	-	-	-	4.4	12.2	3.5	1.9	7.5	3.5	0.2	25.0	5.3	9.0	5.9
Esters																		
Acetic acid, 1-(2-methyltetrazol-5-yl) ethenyl ester (43, 55, 126, 42, 26, 72)	44.771	**2164**	-	-	-	-	-	0.1	-	-	-	-	-	-	-	-	-	-
Butanoic acid, 2-methyl-, 2-methylbutyl ester (43, 70, 56, 46, 85, 55)	12.551	-	1274	-	-	-	-	-	-	-	0.3	-	-	-	-	-	-	-
**Butanoic acid, 3-methyl-,** **3-methylbutyl ester**	13.297	-	1312	-	-	-	-	0.1	-	2.1	3.3	-	0.4	-	-	-	2.3	0.5
**Dichloroacetic acid, 4-hexadecyl ester**	26.944	1591	-	1.4	-	-	-	-	-	-	-	-	-	-	-	-	-	-
**5-Dodecen-1-ol, acetate, (Z)-**	32.178	1753	-	19.9	-	-	-	-	-	-	-	-	-	-	-	-	-	-
**Hexadecanoic acid, methyl ester**	46.421	**2221**	2223	-	-	0.3	0.5	-	-	-	-	-	-	-	0.7	-	-	-
**Hexadecanoic acid, 15-methyl-,** **methyl ester**	52.424	2438	-	-	-	-	-	-	-	-	-	-	-	-	-	0.2	-	-
**Octadecanoic acid, methyl ester**	52.532	**2440**	2417	-	-	0.2	0.7	-	-	-	-	-	-	-	1.6	-	0.2	-
**9-Octadecenoic acid (Z)-, methyl ester**	46.027	2207	2430	-	-	-	0.5	-	-	-	-	-	-	-	-	-	-	-
**10-Octadecenoic acid, methyl ester**	52.928	2455	-	-	-	0.5	0.9	-	-	-	-	-	-	-	2.7	0.4	0.4	-
12,15-Octadecadienoic acid, methyl ester (81, 67, 55, 41, 68, 82)	54.272	2503	-	-	-	-	0.2	-	-	0.3	-	-	-	-	-	-	-	-
2-Phenylethyl acetate	33.237	**1787**	1777	0.4	-	-	-	-	-	-	-	-	-	-	-	-	0.1	0.6
**Propanoic acid, 2-methyl-, 3-hydroxy-2,4,4-trimethylpentyl ester**	35.558	1860	-	-	-	-	-	-	-	-	0.2	0.3	-	-	-	-	-	-
2-Propenoic acid, 3-phenyl-, pentyl ester (148, 131, 103, 147, 41, 149)	66.841	-	-	-	-	-	-	-	-	-	0.2	-	-	-	-	-	-	-
11-Tetradecen-1-ol, acetate, (Z)- (43, 55, 67, 41, 70, 68)	6.825	-	2137	-	-	-	-	-	-	-	3.4	-	-	-	-	-	-	2.1
**2,2,4-Trimethyl-1,3-pentanediol diisobutyrate**	35.840	1864	-	-	-	0.2	0.2	-	-	-	1.5	1.8	-	-	-	-	-	-
Aldehydes																		
Furfural (39, 96, 95, 57, 29, 38)	20.040	-	1432	-	-	-	-	1.3	-	-	-	-	-	-	-	-	-	-
**2-Hexenal**	9.667	-	1225	-	6.6	-	-	-	-	-	-	-	-	-	-	-	-	-
Nonanal	21.166	**1403**	1395	-	-	-	-	-	0.1	-	-	-	-	-	-	-	-	-
5-methyl furfural (110, 53, 109, 27, 39, 41)	24.344	1511	1560	-	-	-	-	0.3	-	-	-	-	-	-	-	-	-	-
Ketones																		
Cyclopentanone, 2-methyl- (45, 55, 28, 43, 42, 41)	12.529	-	-	-	-	-	-	-	-	-	-	-	0.2	-	-	-	-	-
3,4-Dimethyl-2-pentanone (43, 55, 71, 28, 114, 41)	43.604	2124	-	-	-	-	-	0.1	-	-	-	-	-	-	-	-	-	-
2,6-Dimethyl-6-nitro-2-hepten-4-one (83, 55, 43, 29, 27, 39)	32.005	1748	-	-	0.2	-	-	-	-	-	-	-	-	-	-	-	-	-
**4-Heptanone, 3-methyl-**	26.480	1576	-	-	0.1	-	-	-	-	-	-	-	-	-	-	-	-	-
**5-Hepten-3-one, 5-methyl-**	14.312	-	-	-	-	-	-	-	-	-	-	-	0.3	-	-	-	-	-
**3-Octanone**	11.460	-	1205	-	-	-	-	-	-	-	-	-	4.2	-	-	-	-	-
2-Pentanone, 4-hydroxy-4-methyl- (43, 59, 56, 42, 41, 207)	15.961	-	1352	-	-	-	-	-	-	-	-	-	0.2	-	-	-	-	-
**4H-Pyran-4-one, 2,3-dihydro-3,5-dihydroxy- 6-methyl-**	47.988	**2278**	2274	-	-	-	-	0.2	-	-	-	-	-	-	-	-	-	-
Alcohols																		
**1,4-Butanediol**	37.292	1915	-	-	-	-	-	-	-	-	-	-	-	-	0.5	-	-	-
**1-Butanol, 2-methyl-**	9.887	-	1201	-	-	-	-	29.9	-	-	-	12.1	-	1.5	-	-	-	-
**1-Butanol, 3-methyl- (impure)**	9.577	-	1210	-	-	-	-	-	13.7	17.5	38.5	-	49.4	-	36.3	4.4	42.9	26.9
2-Buten-1-ol, 3-methyl- (71, 41, 43, 29, 39, 27)	14.530	-	1301	-	-	-	-	0.2	-	-	-	-	-	-	-	-	-	-
**1-Decanol**	32.153	**1753**	1760	-	-	-	-	-	-	-	-	-	-	0.2	-	-	-	-
1,1-Dimethyl-3-chloropropanol (59, 43, 41, 31, 27, 28)	12.889	-	-	-	-	-	-	-	-	-	-	-	0.2	-	-	-	-	-
Ethanol, 2-(2-ethoxyethoxy)- (45, 31, 59, 29, 72, 27)	26.644	1582	1615	-	-	-	-	-	-	0.2	-	-	-	-	-	-	-	-
2-Hepten-3-ol, 4,5-dimethyl- (71, 43, 32, 55, 29, 27)	20.468	-	-	-	-	-	-	0.2	-	-	-	-	-	-	-	-	-	-
6-Hepten-1-ol, 3-methyl- (55, 71, 73, 43, 67, 41)	54.185	2501	-	-	-	-	-	-	-	-	-	-	-	-	0.6	-	-	-
1-Hexanol	15.314	-	1356	-	8.6	-	-	0.2	0.3	0.3	-	0.6	-	-	-	-	-	-
1-Hexanol, 3-methyl- (56, 55, 70, 32, 69, 26)	24.406	1515	1413	-	-	-	-	-	-	-	0.2	-	-	-	-	-	-	-
**2-Hexen-1-ol, (E)-**	17.570	-	1403	-	17.5	-	-	-	-	-	-	-	-	-	-	-	-	-
**2-Hexen-1-ol, (Z)-**	17.948	-	1380	-	0.2	-	-	-	-	-	-	-	-	-	-	-	-	-
3-Hexen-1-ol, (Z)- (41, 67, 55, 27, 29, 82)	15.715	-	1373	-	4.5	-	-	-	-	0.5	-	0.3	-	-	-	-	-	-
3-Methyl-hepta-1,6-dien-3-ol (71, 43, 28, 55, 57, 29)	20.409	-	-	-	-	-	-	-	-	-	-	0.3	-	-	-	-	-	-
4-Methyl-1-heptyn-3-ol (43, 71, 55, 27, 82, 41)	43.636	2126	-	-	-	-	-	-	-	-	-	-	-	0.1	-	-	-	-
9,12-Octadecadien-1-ol (67, 81, 95, 55, 82, 96)	36.871	1901	-	0.3	-	-	-	-	-	-	-	-	-	-	-	-	-	-
(5Z)-Octa-1,5-dien-3-ol (57, 70, 41, 29, 55, 27)	21.335	1412	-	-	-	-	-	-	-	-	-	0.2	-	-	-	0.2	-	-
1,7-Octadien-3-ol, 2,6-dimethyl- (71, 43, 55, 41, 27, 39)	44.471	2154	-	-	-	-	-	0.8	0.8	1.1	0.7	1.6	0.8	16	-	1.4	0.6	1.1
**1,5,7-Octatrien-3-ol, 3,7-dimethyl-**	25.870	**1558**	1613	-	0.8	-	-	-	-	-	-	-	-	-	-	-	-	-
**1-Octen-3-ol**	19.472	-	1447	-	-	-	-	-	-	0.3	-	-	0.7	-	-	0.4	0.2	0.2
Z-5-octen-3-ol (59, 71, 70, 28, 55, 41)	18.361	-	-	-	-	-	-	-	-	-	-	-	0.1	-	-	-	-	-
**7-Octen-4-ol**	19.975	-	1453	-	-	-	-	-	-	-	-	0.4	-	-	-	-	-	-
1-Octyn-3-ol (43, 71, 41, 28, 55, 82)	43.612	2125	-	-	-	-	-	-	-	-	-	0.2	-	-	-	-	-	-
1-Penten-3-ol, 4-methyl- (57, 41, 70, 39, 66, 82)	21.378	1414	-	-	-	-	-	-	-	0.4	-	-	-	-	-	-	-	-
Acids																		
Benzoic acid	52.562	**2443**	2444	-	-	-	-	-	-	0.2	-	0.3	-	0.2	-	-	-	-
**n-Hexadecanoic acid**	65.080	-	2880	-	-	-	-	-	-	0.3	0.2	0.3	-	-	-	-	-	-
2-Methylbutanoic acid	28.485	**1638**	1641	-	-	-	-	0.1	-	-	-	-	-	-	-	-	-	-
9,12,15-Octadecatrienoic acid, (ZZZ)- (79, 55, 41, 67, 93, 95)	34.191	1818	3292	1.2	-	-	-	-	-	-	-	-	-	-	-	-	-	-
Straight hydrocarbons																		
**6-Dodecyne**	24.713	1522	-	0.2	-	-	-	-	-	-	-	-	-	-	-	-	-	-
**9-Eicosene, (E)-**	37.632	1926	-	1.4	-	-	-	-	-	-	-	-	-	-	-	-	-	-
**Heptadecane**	30.150	1689	1700	3.0	-	-	-	-	-	-	-	-	-	-	-	-	-	-
**8-Heptadecene**	29.916	1682	1718	-	-	-	-	-	-	-	-	0.3	-	-	-	-	-	-
5-Heptadecene, 1-bromo- (91, 41, 55, 67, 81, 109)	35.472	1857	-	0.7	-	-	-	-	-	-	-	-	-	-	-	-	-	-
Heptane, 1-fluoro- (75, 69, 41, 72, 28, 105)	21.693	1424	-	-	-	-	-	-	-	-	0.2	-	-	-	-	-	-	-
**Hexadecane**	26.277	1570	1600	1.77	-	-	-	-	-	-	-	-	-	-	-	-	-	-
2-Hexene, 3,5,5-trimethyl- (57, 41, 70, 29, 27, 42)	21.405	1415	-	-	-	-	-	-	-	-	-	-	0.2	-	-	-	-	-
**Nonadecane**	37.199	1912	1900	0.7	-	-	-	-	-	-	-	-	-	-	-	-	-	-
**9-octadecene (E)-**	30.799	1708	-	31.8	-	-	-	-	-	-	-	-	-	-	-	-	-	-
**9-Octadecyne**	28.461	1637	-	0.2	-	-	-	-	-	-	-	-	-	-	-	-	-	-
**Pentadecane**	23.875	1495	1500	19.6	-	-	-	-	-	-	-	-	-	-	-	-	-	-
**5-Tetradecene, (E)-**	19.268	-	-	0.35	-	-	-	-	-	-	-	-	-	-	-	-	-	-
**Tridecane**	13.703	-	1300	0.49	-	-	-	-	-	-	-	-	-	-	-	-	-	-
Others																		
**Butyrolactone**	25.260	**1612**	1617	-	-	-	-	-	0.2	-	0.3	0.5	0.5	1.1	-	-	-	-
**gamma-Butyrolactone**	26.124	1567	-	-	-	-	-	-	-	0.3	0.3	-	-	-	-	-	-	-
Dihydroxyacetone (31, 29, 33, 43, 32, 44)	42.282	2080	-	-	-	-	-	0.2	-	-	-	-	-	-	-	-	-	-
**2,5-divinyl-2-methyl-tetrahydrofuran**	7.724	-	-	-	-	-	-	-	-	-	2.4	0.3	-	-	-	-	-	0.7
Furan, 3-(4-methyl-3-pentenyl)- (41, 53, 69, 81, 27, 150)	18.103	-	1431	-	-	0.5	-	-	-	-	-	-	-	-	-	-	-	-
2-Furancarboxaldehyde, 5-(hydroxymethyl)-	54.536	**2513**	2513	-	-	-	-	4.2	-	-	-	-	-	-	-	-	-	-
Geranyl bromide (69, 41, 79, 121, 39, 81)	13.693	-	-	-	-	-	-	-	-	-	-	-	0.5	-	-	-	-	-
**1,4-Hexadiene,** **5-methyl-3-(1-methylethylidene)-**	16.338	-	-	-	-	3.7	3.6	1.5	1.5	1.4	0.5	1.0	4.2	0.3	-	0.2	-	-
Hexanoyl chloride (43, 52, 41, 28, 93, 26)	11.421	-	-	-	-	-	-	0.7	-	-	-	-	-	-	-	-	-	-
1-Imidazol-1-yl-2,2-dimethylpropan-1-one (57, 41, 85, 69, 68, 40)	17.392	-	-	-	-	-	-	-	-	0.3	-	-	-	-	-	-	-	-
Isoamyl cinnamate (148, 131, 103, 147, 41, 149)	66.838	-	-	-	-	-	-	-	-	-	-	0.4	-	-	-	-	-	-
1,3-Pentadiene, 5-(2,2-dimethylcyclopropyl)-2,4-dimethyl-, (Z or E)- (41, 121, 69, 105, 39, 79)	17.358	-	-	-	-	-	-	0.3	-	-	-	-	-	-	-	-	-	-

* Retention Indices were calculated according to van Den Dool and Kratz (1963); ** Retention Indices according to https://webbook.nist.gov/, Van Den Dool and Kratz RI for polar column and, if possible, temperature ramp identical or at least similar to this analysis; *** Mass spectral fragments for unconfirmed compounds in descending order.

**Table 2:** Plants material used in the experiment.

Species	Gender	Genotype	Place of Plants Origin
*A. chinensis* var. *deliciosa*	male	seedling	Lublin Botanical Garden, Poland
*A. polygama*	male	seedling	USDA Germplasm Repository, Corvallis, US
*A. kolomikta*	male	‘Adam’	“Clematis the Source of Good Climbers” Nursery, Poland
*A. kolomikta*	female	‘Tallinn’	Private collection, Poland
*A. arguta*	male	‘Haya Kume’	USDA Germplasm Repository, Corvallis, US
*A. arguta*	male	‘Joker’	Own selection
*A. arguta*	male	‘Nostino’	Haeberli Nursery, Switzerland
*A. arguta*	male	‘Rot’	Werner Merkel, Germany
*A. arguta*	male	‘Rubi’	Own selection
*A. arguta*	male	‘Weiki’	“Clematis the Source of Good Climbers” Nursery, Poland
*A. arguta*	male	F7	Own selection
*A. arguta*	female	‘Ananasnaya’(= ‘Anna’)	Fachhochschule Weihenstephan, Freising, Germany
*A. arguta*	female	‘Bingo’	Own selection
*A. arguta*	female	‘Geneva’	“Clematis the Source of Good Climbers” Nursery, Poland
*A. arguta*	female	‘Weiki’	“Clematis the Source of Good Climbers” Nursery, Poland

## References

[1] Li X., Li J., Soejarto D.D. (2008). Advances in the study of the systematics of *Actinidia* Lindley. Front. Biol. China.

[2] Ferguson A.R. (2016). Botanical Description.

[3] Pinto T. (2018). Kiwifruit, a botany, chemical and sensory approach a review. Adv. Plants Agric. Res..

[4] Chesoniene L., Daubaras R., Viskelis P. (2004). Biochemical composition of berries of some Kolomikta kiwi (*Actinidia Kolomikta*) cultivars and detection of harvest maturity. Acta Hortic..

[5] Latocha P., Łata B., Stasiak A. (2015). Phenolics, ascorbate and the antioxidant potential of kiwiberry vs. common kiwifruit: The effect of cultivar and tissue type. J. Funct. Foods.

[6] Leontowicz M., Leontowicz H., Jesion I., Bielecki W., Najman K., Latocha P., Park Y.-S., Gorinstein S. (2016). Actinidia arguta supplementation protects aorta and liver in rats with induced hypercholesterolemia. Nutr. Res..

[7] Latocha P. (2017). The Nutritional and health benefits of Kiwiberry (*Actinidia arguta*)—A review. Plant Foods Hum. Nutr..

[8] Rodrigues F., Delerue-Matos C., Rodrigues F. (2020). Bioactivity, phytochemical profile and pro-healthy properties of *Actinidia arguta*: A review. Food Res. Int..

[9] Kim Y.K., Kang H.J., Lee K.T., Choi J.G., Chung S.H. (2003). Anti-inflammation activity of *Actinidia polygama*. Arch. Pharmacal Res..

[10] Testolin R. (1991). Male density and arrangement in kiwifruit orchards. Sci. Hortic..

[11] Costa G., Testolin R., Vizzotto G. (1993). Kiwifruit pollination: An unbiased estimate of wind and bee contribution. N. Z. J. Crop. Hortic. Sci..

[12] Tiyayon C., Strik B.C. (2003). Flowering and fruiting morphology of hardy kiwifruit, *Actinidia arguta*. Acta Hortic..

[13] Fraser L., Mcneilage M. (2016). Reproductive Biology.

[14] Kawagoe T., Suzuki N. (2004). Cryptic dioecy in *Actinidia polygama*: A test of the pollinator attraction hypothesis. Can. J. Bot..

[15] Reisenman C.E., Riffell J.A., Bernays E.A., Hildebrand J.G. (2010). Antagonistic effects of floral scent in an insect–plant interaction. Proc. R. Soc. B: Boil. Sci..

[16] Dötterl S., Burkhardt D., Weißbecker B., Jürgens A., Schütz S., Mosandl A. (2006). Linalool and lilac aldehyde/alcohol in flower scents. J. Chromatogr. A.

[17] Twidle A.M., Mas F., Harper A.R., Horner R.M., Welsh T.J., Suckling D.M. (2015). Kiwifruit flower odor perception and recognition by honey bees, *Apis mellifera*. J. Agric. Food Chem..

[18] Twidle A.M., Barker D., Seal A.G., Fedrizzi B., Suckling D.M. (2018). Identification of floral volatiles and pollinator responses in kiwifruit cultivars, *Actinidia chinensis* var. *chinensis*. J. Chem. Ecol..

[19] Stasiak A., Latocha P., Drzewiecki J., Hallmann E., Najman K., Leontowicz H., Leontowicz M., Łata B. (2019). The choice of female or male parent affects some biochemical characteristics of fruit or seed of kiwiberry (*Actinidia arguta*). Euphytica.

[20] Tatsuka K., Suekane S., Sakai Y., Sumitani H. (1990). Volatile constituents of kiwi fruit flowers: Simultaneous distillation and extraction versus headspace sampling. J. Agric. Food Chem..

[21] Samadi-Maybodi A., Shariat M.R., Zarei M., Rezai M.B. (2011). Headspace analysis of the male and female flowers of kiwifruit grown in Iran. J. Essent. Oil Res..

[22] Twidle A.M., Suckling D.M., Seal A.G., Fedrizzi B., Pilkington L.I., Barker D. (2017). Identification of in situ flower volatiles from kiwifruit (*Actinidia chinensis* var. *deliciosa*) cultivars and their male pollenisers in a New Zealand orchard. Phytochemistry.

[23] Matich A.J., Young H., Allen J.M., Wang M.Y., Fielder S., Mcneilage M., Macrae E.A. (2003). *Actinidia arguta*: Volatile compounds in fruit and flowers. Phytochemistry.

[24] Bertrand C., Comte G., Piola F. (2006). Solid-phase microextraction of volatile compounds from flowers of two *Brunfelsia* species. Biochem. Syst. Ecol..

[25] Matich A.J., Bunn B., Comeskey D., Hunt M., Rowan D.D. (2007). Chirality and biosynthesis of lilac compounds in *Actinidia arguta* flowers. Phytochemistry.

[26] Kohlmünzer S. (2007). Farmakognozja.

[27] Huang H. (2014). The Genus Actinidia. A World Monograph.

[28] Xu M., Jin Z., Yang Z., Rao J., Chen B. (2020). Optimization and validation of in-situ derivatization and headspace solid-phase microextraction for gas chromatography-mass spectrometry analysis of 3-MCPD esters, 2-MCPD esters and glycidyl esters in edible oils via central composite design. Food Chem..

[29] Howlett B.G., Read S., Jesson L., Benoist A., Evans L., Pattemore D.E. (2017). Diurnal insect visitation patterns to ‘Hayward’ kiwifruit flowers in New Zealand. N. Z. Plant. Prot..

[30] Cordeiro G.D., Pinheiro M., Dötterl S., Alves-Dos-Santos I., Dafni A. (2016). Pollination of Campomanesia phaea (Myrtaceae) by night-active bees: A new nocturnal pollination system mediated by floral scent. Plant. Biol..

[31] Krug C., Cordeiro G.D., Schäffler I., Silva C.I., Oliveira R., Schlindwein C., Dötterl S., Alves-Dos-Santos I. (2018). Nocturnal bee pollinators are attracted to guarana flowers by their scents. Front. Plant. Sci..

[32] Henning J.A., Peng Y.-S., Montague M.A., Teuber L.R. (1992). Honey bee (Hymenoptera: Apidae) behavioral response to primary alfalfa (Rosales: Fabaceae) floral volatiles. J. Econ. Èntomol..

[33] Lukas K., Harig T., Schulz S., Hadersdorfer J., Dötterl S. (2019). Flowers of European pear release common and uncommon volatiles that can be detected by honey bee pollinators. Chemoecology.

[34] Aceves-Chong L., Cruz-López L., Sánchez-Guillén D., Grajales-Conesa J. (2018). Differences in volatile composition and sexual morphs in rambutan (*Nephelium lappaceum* L.) flowers and their effect in the *Apis mellifera* L. (Hymenoptera, Apidae) attraction. Rev. Bras. Èntomol..

[35] Van Den Dool H., Kratz P.D. (1963). A generalization of the retention index system including linear temperature programmed gas-liquid partition chromatography. J. Chromatogr. A.

